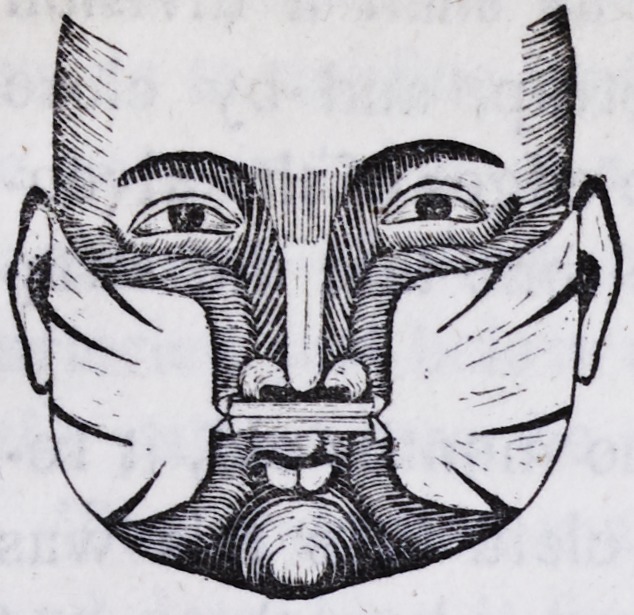# Hare-Lip, and Its Treatment

**Published:** 1844-06

**Authors:** S. P. Hullihen

**Affiliations:** Wheeling, Va.


### ARTICLE III.

*Hare-lip, and its Treatment.* By DR. S. P. HULLIHEN, Wheeling, Va.

HARE-LIP is the most common of all congenital deformities, and the most painfully offensive to the eye. This deformity may be divided into two varieties, the single and the double. The single consists in a division or fissure of the lip, corresponding, most generally, with one of the nostrils. The double differs only from the single in having two fissures, one corresponding with each nostril. A small portion of lip is therefore left hanging from the base of the septum nasi. This portion of lip is shorter and thinner than the lip on each side. The edges and angles of the lip, formed by the fissure, are always rounded, and this rounding comprehends the only loss of substance occasioned by the deformity. Both varieties of hare-lip are most frequently accompanied with a cleft or fissure extending through the alveolar arch, the roof of the mouth, and the soft palate. With this complication, the deformity is greatly increased; the nose is more or less drawn to one side, the nostril over the cleft in the jaw is spread out and depressed, and one end of the divided alveolar arch frequently projects forward, while the fissure in the lip is thrown wide apart, and the jaws and tongue are frightfully exposed, imparting a most hideous aspect. In such cases of hare-lip, the deformity may be greatly relieved, but not removed, unless operated upon at the most favorable period of life, and then only when the operation is preceded by proper preparatory treatment.

*The age most favorable for the operation.*—The most favorable age for the complete removal of the deformity of hare-lip, all concede to be in infancy; but there is some difference of opinion as to the time most proper for the operation during this period. It has been urged by some writers, that the first four or five months, being before dentition usually commences, is the most proper time, as the child is then most easily managed, the deformity most effectually removed, and the constitutional effects of an operation less to be dreaded, than at any other period. This opinion, however, has been ably combatted, and by high authority, on the ground that young infants bear the loss of blood badly; that the pins are liable to tear out in the lip of so tender a subject; and that the consequent irritation, attending such an operation, often produces convulsions, and even death. They therefore contend that the most proper time for the operation is from one to three years after birth.

Fearful as these objections to early operations may appear, there is, in reality, no force in them when separately examined, and in connection with such preparatory treatment as most cases of hare-lip, in young infants, imperiously demand.

That an infant can sustain but a small loss of blood compared with an adult is an important fact, and one that should be most carefully remembered. But it is strange that this objection should be urged against operating on infants for the cure of hare-lip, when the bleeding in such cases is but trifling, and that, under such entire control! While the edges of the lip are being pared off, the pins inserted, and the ligatures applied, simple pressure upon the external maxillary arteries, or upon those of the lip, by seizing the lip between the thumb and finger, is all that is required to control the hæmorrhage to the most limited degree. If such simple but necessary precautions be neglected in operating, and the life of an infant be endangered or even destroyed from the loss of blood, the fault is surely not in the early operation, but in the manner of its performance. This objection, therefore, is of no valid import. That the lip of a young infant is tender, and that the pins may sometimes slough or tear out comparatively easy, when greatly stretched or dragged together over the projecting end of a cleft alveolar process, there can be no doubt. But if the division in the jaw be first closed, and its natural arch restored, the interspace in the lip would be so small that no such stretching or dragging together of the lip would be required, and sloughing or tearing out of the pins could not then of course occur.

If pins, therefore, slough or tear out in some bad cases of hare-lip in young infants, the fault must not be attributed to the tenderness of the lip, but to the want of proper preparatory treatment; a very important difference.

That local irritation is a common cause of convulsions in infants, is fully proved by their more frequent occurrence during dentition than at any other period of life; and that the irritation attending a great stretching or dragging together of the lip will likewise produce convulsions, in some infants, cannot be denied. Yet there is a grade of irritation necessary to produce these results, and that grade can only exist, in this operation, from too great a tension of the lip, and this tension from a cleft in the alveolar process, which cleft can always be closed before an operation should be performed, thereby removing at once the necessity of any tension, the source of irritation, and the cause of convulsions. The objections, then, to early operations in infants, for the cure of this deformity, appear to have been based on certain effects which were attributed, as shown, to wrong causes.

The operation on infants, for the cure of hare-lip, before the period of dentition has commenced, is more easily accomplished, presents more facilities for the complete removal of the deformity, and is less fraught with danger to the infant, and to the success of the operation, than at any other period.

The infant, before dentition commences, has no fears of an operation, and therefore makes no resistance nor struggles, except those excited by the painful manipulations of the operator; and these being but momentary, and the child easily managed, the lip can be more satisfactorily prepared, and elegantly adjusted, than can possibly be accomplished on a child a few more months advanced in life. This circumstance, alone, is of much importance.

The facilities for the more complete removal of the deformity of hare-lip before dentition commences, are very great and very important, where the deformity occurs in connection with a cleft in the alveolar and palatine arches. The bones of the face, at this period, being in a soft and cartilaginous state, can readily be brought into any desired position. The cleft in the alveolar arch can therefore be closed, its projections connected, its arch restored, which is as indispensably necessary to the complete removal of the deformity, as a perfect adaptation of the lip. In addition to all this, nature makes a greater and more successful effort to restore all deficiencies at this period of life than at any other.

The operation before dentition commences, is likewise less fraught with danger to the infant and to the success of the operation than at any other period. Less fraught with danger to the infant, because the irritation consequent upon the operation can be rendered harmless, in all cases, by proper preparatory treatment; and because an infant is much less subject to convulsions before dentition, than after this process has commenced; neither is it liable to a host of other symptomatic diseases that so frequently accompany dentition, endangering and destroying life, independent of the consequences that might be added by the effects of an untimely operation. It is less dangerous to the success of an operation, because, at this time of life, an infant sleeps more than at any other, is less disposed to fret and cry, is less liable to disturb the lip and dressings with its hands, and is far more easily managed in every way that tends to the security and successful termination of a case.

I am, therefore, decidedly in favor of early operations on infants, for the cure of hare-lip. I have operated on thirteen cases before dentition had commenced, three infants of this number were only four weeks old; and I have yet to witness the first untoward event, or the slightest unfavorable indication resulting to an infant from the operation.

*Preparatory Treatment.*—Preparatory treatment is applicable in all cases of hare-lip during infancy, where the deformity is accompanied with a cleft of the alveolar and palatine arches. It consists in restoring the alveolar arch to its proper form, before the operation for the cure of hare-lip is attempted.

A cleft of the alveolar and palatine arches, like that of the lip, is a congenital separation of the parts, with but little if any loss of substance. Its connection, therefore, with hare-lip greatly increases the deformity of the whole countenance. The edges of the lip formed by the fissure are always carried apart as much farther than is usual in simple cases of hare-lip, as the cleft may be wide in the alveolar arch. The nostril over the cleft is likewise stretched out and depressed; a projection of the alveolar process frequently occurs, and the face is always very perceptibly widened, all resulting from the cleft in the jaw, and all increasing or diminishing in deformity, in proportion as the cleft may vary in width.

To unite the edges of hare-lip, where this complication of the deformity exists, is always more or less difficult, and sometimes even impossible, and when accomplished will not restore the form of the nostril, correct the projections of the alveolar process, nor relieve the unseemly width of the face, except in a very limited degree. But closing the cleft in the alveolar arch corrects, at once, all these irregularities, and at the same time approximates the edges of the lip so closely that they may be most admirably united, without the least danger to the infant or to the success of the operation. It is upon these grounds that the utility and importance of preparatory treatment is urged.

The closure of the cleft in the alveolar arch may be effected in a variety of ways; but the most simple, and at the same time the most effectual, may be accomplished solely by the use of the adhesive strap, properly applied upon the cheeks.

The cartilaginous state of the bones in early infancy requires but little force to bring them into any desired position. But in removing the deformity of a cleft alveolar arch, a force of a two-fold nature is often required, both to bring the edges together and at the same time compress any projections of the alveolar process which may exist. In the proper application of the adhesive strap may be found this happy combination.

The form of the strap which I have usually employed for this purpose is represented in the following cut. It should be left as large at each end as the size of the cheek will permit, and slitted at different places, so that it may adhere smoothly and firmly. The part required to pass over the lip should be somewhat less than half an inch in width, the edges of this part being doubled over and fastened together, in order to give it the necessary strength and stiffness.

The strap may be applied in the following manner: after being properly warmed, one end should be quickly and well adhered to the cheek of one side, then, pressing both cheeks forward, and passing the strap over the upper lip, close to the nose, it should be adhered in like manner to the cheek of the other side. By thus confining the cheeks forward, a force is obtained and exerted upon the jaw, sufficiently great to close in a few weeks the widest cleft of the alveolar arch, and at the same time to correct any projections of its process. The strap should be kept perfectly tense. It is therefore necessary to tighten it every day or two, which may be done by cutting a small portion out of the narrow part, and then sewing it together, without disturbing its adhesions to either cheek. In this way, the same strap will last for several days, and is so easily tightened that its management may be safely entrusted to the parents of the child. As the wearing of the strap never excoriates the parts, nor produces the least pain to the infant, however young, it is advisable to apply it as soon after birth as possible, as a cleft in the alveolar arch is more easily closed at this period than at any other; and as the strap is always of very great assistance to the infant in taking its food. In cases of simple hare-lip, without any cleft in the alveolar arch, the use of the strap will enable the child to nurse at the breast with but little if any difficulty.

In the summer of 1839, I was requested to see an infant that had been born a day or two before, with hare-lip, the fissure extending into the nostril, but without any deformity of the jaw. I immediately applied the strap, with the view of enabling the child to nurse at the breast, and the experiment was perfectly successful. The child could at once seize and retain the nipple in its mouth, and soon learned to suck without any difficulty. Since then, I never have had an opportunity of repeating the experiment, except on an infant that had previously acquired the habit of receiving its food, for a long time, from the spoon. In this case the result was entirely unsuccessful.

The time generally required to close a cleft of the alveolar arch, depends more upon the age of the infant than upon the size of the cleft. In the year 1838, M. H., of this city, requested me to see an infant of his, that had been born the night before, with a hare-lip, and the most extensive division of the alveolar and palatine arches, I ever witnessed. The cleft was nearly an inch in width, causing such deformity of the face as such a division can only produce. I at once applied the strap, and by close attention to the case succeeded in bringing the edges of the alveolar process together, in three weeks from the time that the strap was first applied.

In another case where the child was nine months old, it required eight weeks to close a much smaller cleft. As this was the oldest child, I was ever called upon to treat, where the use of the strap would have been of the least advantage, I have no means of determining the length of time it would require to close a cleft in a child of one or two years of age. It generally requires from four to six weeks to close the cleft in infants under five months old.

As soon as the cleft edges of the alveolar arch, are brought together so as to touch each other in the slightest manner, the operation for the cure of hare-lip may be properly performed. The union of the lip in all such cases has the effect of completing the closure of the cleft in the alveolar arch. The treatment of the cleft in the roof of the mouth and soft palate, must now be abandoned, until the patient becomes more advanced in life, and may, perhaps, form the subject of some future paper.

*Operation for the Cure of Hare-lip.*—The general principles of the operation for the cure of hare-lip consist, first, in reducing the edges of the lip to a simple incised wound; then, in inserting the needles so that the edges of the wound may be brought evenly together; then, in confining the edges together until they are firmly healed. But, in addition to these general indications, a particular plan should be adopted in each operation, with the view of making a well formed lip, and this plan must be made with a strict reference to the peculiarities of the case, and be carefully and plainly marked out upon the lip before the operation is commenced.

The instruments necessary for the operation are, a scalpel, for detaching the lip from the jaw, a pair of dressing forceps to hold the lip, a pair of scissors or a bistoury, to pare off the edges, three or four long spear-pointed steel needles, several silk ligatures, a pair of cutting nippers to remove the ends of the needles, and a sponge or two.

The patient, if a child, may be first wrapped up in a long towel, so as to confine its legs and arms securely, and then be placed on a narrow table, in a reclining position, and firmly held by assistants, one of them making pressure upon its external maxillary arteries, just below and forward of the masseters. If an adult, the patient may be seated upon a chair.

The operation may be commenced by turning the lip upwards, and detaching it from the jaw, to such extent as the case may require. If the interspace in the lip is small, little or no dissection will be necessary; if large, a very free dissection is always required, extending along the jaw, and up under the wing of the nostril, (particularly if it is spread out and depressed,) until the detached parts give way sufficiently to permit the fissure of the lip to be easily closed, and the form of the nostril greatly improved. This part of the operation being finished, the next step is:

*To pare off the edges of the lip.*—This should be done in such a manner, that when brought together the lip will have a natural length, its hanging edge a proper form, and the mucous membrane covering this edge a corresponding width. To effect this, some cases may require one side of the lip to be pared off straight, and the other side concave. Sometimes both sides may be pared off straight; in other cases, both sides concave. In some cases, a broad portion of the lip may be removed on one side, and a very small portion from the other; but, in *all cases, as much of the lip as may be rounded* must be invariably removed. It is always better that too much of the lip should be taken away than too little.

Having determined upon a plan, and carefully drawn upon the lip the lines to be exactly followed in paring off the edges, the lip may be seized with a pair of forceps by the part to be removed, then putting it slightly on the stretch, the edges may be cut away as desired by a stroke or two with the scissors or bistoury, as the operator may prefer. As each edge is pared away, an assistant should control the bleeding, by laying hold of the lip, and compressing it between his thumb and finger. The edges of the lip being removed on both sides of the fissure, the next step is:

*To insert the needles.*—This should be done at equal distances, taking a sufficient hold of the lip to prevent them from tearing out, and placing them in such a manner that the edges of the lip may be brought evenly together, both sides corresponding in every particular.

The lower needle should be always inserted first, and always in the red of the lip, and at least three lines back from the pared off edge. Then push it a little obliquely from below upwards, and from without inwards, until the point appears in the pared off edge, a little above the mucous membrane; then turn the point of the needle rather downwards, and introduce it into the other edge of the lip, precisely opposite the point where it came out first; then push it from above obliquely downwards and from within outwards, until the point appears in the red of the lip, as far back from the pared off edge as it may have been entered on the other side. A temporary ligature should now be thrown round the ends of the needle, and secured by a knot. The second needle should be inserted horizontally, and midway between the first needle and the nose, and much nearer the internal than the external surface of the lip. The third and last needle should be inserted as close to the nose as possible, and after the manner of the second. All the needles being inserted, the next step is:

*To apply the ligatures.*—This should be done so that the raw edges of the lip fit closely and neatly, without being pressed together unnecessarily tight. There is always as much danger of excessive suppuration about the needles, and of the needles sloughing out from too great a tightness of the ligatures, as from the greatest drag upon the lip, however wide the fissure may be.

The edges of the lip being properly fitted together, a short ligature may be thrown around the middle needle, and tied; then cutting away the temporary ligature from the lower needle, and adjusting the edges of the lip as they should be confined, a ligature of two feet in length may be passed round the ends of the needles, and carried backwards and forwards, crossing midway of each turn, until it is entirely consumed. A temporary ligature should then be thrown around the upper needle and secured, and the ligature on the middle needle cut away, and a long one applied in the same manner as that upon the lower needle; and then another, in like manner, upon the upper needle. All the ligatures being now applied, it only remains to cut off the ends of the needles close to the ligatures, and the operation will be finished.

No strips of adhesive plaster, nor bandages of any kind, should be applied over the lip, with the view of supporting it, until after the needles are removed. Such dressings always do more injury than benefit, by confining the secretions, and by their pressure upon the needles causing much unnecessary pain.

The needles may be removed the third or fourth day after the operation, depending entirely upon the amount of suppuration that may exist at the time, about the needles. The cheeks being held forward by an assistant, the upper needle may be seized with a pair of plyers, and after turning it round upon its axis, it should be slowly and gently withdrawn from the lip. Then removing the middle and lower needles in like manner, the assistant still holding the cheeks forward; an adhesive plaster should be applied in the same manner as described in the preparatory treatment, and in such a way as to prevent the slightest pull of the muscles upon the new adhesion of the lip. After four or five days the adhesion of the lip becomes sufficiently firm, and the wearing of the strap may be discontinued.

*The double hare-lip* should be treated upon the same principles in every respect, as the single. The only difference in the operation consists in cutting the small portion of lip that hangs in all these cases from the septum nasi, into a V, or wedge shape, so that it may be fitted neatly between the edges of the lip, in the upper part of the fissure. The lower and middle needles should always pass below this wedge-shaped portion, and the upper needle through it. In this way the lip may be brought evenly together and healed, the line of union having the appearance of the letter Y.

In both varieties of hare-lip, cases are frequently presented in adults, in which it becomes necessary to remove irregular teeth, and projections of the alveolar process, before an operation can be performed. In all such cases the arch of the jaw should be made as perfect as possible without any reference to the number of teeth or amount of bone it may be necessary to sacrifice in accomplishing this object. The bone nippers, saw, and tooth forceps, are the instruments usually employed in removing such deformities of the jaw.

In curing hare-lip, it should always be the uncompromising aim of the operator, to remove the deformity as completely as possible, however tedious the process or difficult the operation may be, that is best calculated to effect the purpose. He that can be satisfied with any course of treatment short of this, should never do a patient the injustice to attempt the operation.

### ARTICLE IV.

*Compound Root Forceps.* By DR. S. P. HULLIHEN, Wheeling, Va.

## Figures and Tables

**Figure f1:**